# Characterization of nanofluids using multifractal analysis of a liquid droplet trace

**DOI:** 10.1038/s41598-022-15402-4

**Published:** 2022-06-30

**Authors:** J. Augustyniak, I. Zgłobicka, K. Kurzydłowski, P. Misiak, A. Z. Wilczewska, Jürgen Gluch, Zhongquan Liao, D. M. Perkowski

**Affiliations:** 1 Faculty of Mechanical Engineering, Bialystok University of Technology, 15-351 Białystok, Poland; 2 Faculty of Chemistry, University of Bialystok, 15-245 Białystok, Poland; 3 Fraunhofer Institute for Ceramic Technologies and Systems IKTS, 01109 Dresden, Germany

**Keywords:** Nanoparticles, Mechanical engineering, Characterization and analytical techniques, Imaging techniques, Microscopy

## Abstract

The article presents an innovative approach to the analysis of nanofluids using a nonlinear multifractal algorithm. The conducted research concerned nanofluids prepared from SiO_2_ nanoparticles (~ 0.01 g) suspended in 100 ml of demineralized water and in 100 ml of 99.5% isopropanol. Subsequently, the nanofluids were subjected to conventional characterization methods such as: determination of the contact angle, determination of zeta potential, pH, and particle size analysis. The obtained results show that the prepared nanofluid is stable in terms of agglomeration over time (nanofluid suspension) and properly prepared in terms of dissolving and dispersing powder particles. The authors, analyzing the results of the presented methods for characterizing nanofluids, proposed a multifractal analysis, which allows detailed local descriptions of complex scaling behaviour, using a spectrum of singularity exponents. Nonlinear analyzes show that the use of multifractal algorithm for nanofluids can improve the process of fluid quality analysis and its preparation based on the multifractal spectrum.

## Introduction

Fluid mechanics plays an extremely important role in many areas of industry, life and science. A serious challenge that the current world is facing is improving the quality of fluids, resulting in creating nanofluids–fluids with suspended nanoparticles. Nanofluids, characterized by a significant increase in the properties of the fluid, including thermal conductivity, long-term stability, homogeneity, etc. compared to conventional engineered fluid^1^, are found to serve in a number of engineering applications, for example porous media^2,3^, etc. However, the preparation process of this type of fluid can be challenging (agglomeration is a major problem in nanofluid synthesis^4^) and even more so, the process allowing to verify the quality of the obtained nanofluid. The main methods by which nanofluids are produced are the two-step or one-step method. The one-step method is a process which combines the production of nanoparticles with the synthesis of nanofluids. The nanoparticles are produced by direct method by physical vapour deposition (PVD) technique or condensation nanoparticles from the vapour phase into a flowing low vapour pressure liquid (VEROS) see^5^. The work^6^ is devoted to the developed Direct Evaporation Condensation method that provides excellent control over the size of nanoparticles and produced a stable nanofluid without the use of any additives. The two-step method is the most widely used method for producing nanofluids. It consists in the earlier acquisition of nanoparticles by other methods, and in the second step, the nanopowder is distributed in the liquid using various methods, including magnetic force agitation^7–9^, ultrasonic agitation, high-shear mixing, homogenization^10^, and ball milling.

The stability of the nanofluids is very important for practical reasons. The potential economic and societal impact is related to the role of nanoparticles as special modifiers to fluids of potentially very wide range of applications (heat transfer: engine cooling/vehicle thermal management, boiler flues gas temperature reduction, heat exchanger, microelectronics etc.). In heat transfer applications, the diffusion coefficient of the particles is directly proportional to the temperature of the fluid. A high diffusion coefficient means more particle collisions and a greater chance of particle aggregation. High temperature applications require additional care to suppress nanofluid instability. In drug delivery systems, undesirable particle sedimentation or aggregation can affect the flow of nanofluids through the channel. The stability of nanofluids is strongly dependent on the properties of the suspended particles and base fluid, such as particle morphology, the chemical structure of particles, and alkaline fluid^11^. Another key parameter in obtaining stable nonafluids is the pH control as was mentioned in^12,13^.

The visualization of the particles within a liquid may be difficult to implement from a technical point of view (scale, number of particles, liquid, 3D spaces). In the works^14,15^ several methods recommended for the analysis of nanoparticles can be found. Among them are: electron microscopy (EM), dynamic light scattering (DLS), centrifugal liquid sedimentations (CLS), field flow fractionation (FFF), inductively coupled plasma–mass spectroscopy system (ICP-MS) and many more. Nevertheless, the assessment of the quality of nanoparticles can be carried out considering separation of super-aggregates of nanostructured particles via sonication as well as evaporation process of a nanofluid droplet. Authors in^16^ mention also the change of pH values of the suspension, by usage of surface activator.

These and other treatments reflect the possible preparation procedures and enable the fabrication of a final product with a defined size range of particles uniformly suspended in the fluid. Whatmore, the analysis of images of samples that have undergone the evaporation process (analysis of the trace left by nanopowder) may also provide information about the fluid itself (the flow dynamics in a sessile droplet) as well as the properties of the powder (particle motion and different patterns formed). The aggregation process is different in solution/fluid after evaporation because of intricate re-assembling process between the nanoparticles nanoparticles (i.e. surface atomic defects of the NPs, electronegative interactions, environmental evaporation parameters, etc.). The formation of the pattern of nanoparticles of isodensity water-based nanofluid drops has been the subject of investigations (see^17^). Authors report two different patterns which depend on the evaporation: an o-ring and a continuous nanoparticle flower pattern. The attempt of understanding the effect of the dynamic of fluid and particle motion that allows obtaining various patterns of nanoparticle sessile after evaporation of fluid has been presented in^18^. The three competitive, convective mechanisms have been considered. Based on obtained results, two major patterns have been identified: the o-ring and the petal-like pattern.

Further investigations in this subject^19^ included the effect of nanoparticle size on evaporation and dryout characteristics of nanofluid droplets via the usage of a microfabricated linear heater array. The three main periods occurring in the evaporation process have been noticed: (i) liquid dominant evaporation, (ii) dryout progress, and (iii) formation of nanoparticle stains.

Another type of research that is gaining importance in the field of research is devoted to the nonlinear analysis of nanofluids or their properties, such as: formulating of aggregates, convective heat transfer, critical heat flux, and subcooled pool boiling heat transfer—this research is based on fractal analysis. In the work^20^ authors introduce three novel fractal models which have a good agreement between fractal model predictions and experimental data. Authors of^21^ conducted experiments on the aggregate fractal dimensions and thermal conduction in nanofluids. The usage of fractals gave them information that as aggregates grow the viscosity increases at a faster rate than thermal conductivity making highly aggregated nanofluids unfavorable. On the other hand, in the paper^22^ authors classified the complex stream patterns taking into consideration fractal dimensions.

In this publication characterization results of dried nanofluid droplets are presented. SiO_2_ nanoparticles were dispersed in water and isopropanol using sonication. The nanofluids were subjected to conventional characterization methods such as: determination of the contact angle, determination of zeta potential, pH, and particle size analysis. Afterwards, single drops were left to evaporate on stubs to conduct microscope observations. The obtained scanning electron microscope (SEM) images were subjected to nonlinear multifractal analysis which allowed validation of the quality of the prepared nanofluid considering the size and distribution of the nanoparticles. An additional advantage of the work is an attempt to demonstrate the versatility of using nonlinear multifractal analysis to characterize the quality of prepared nanofluids on the basis of their evaporated droplet samples.

## Results

### Classic approach and results

As can be seen from the results presented in Table S1, the angles measured on each of the drops in relation to the obstruction (left angle–right angle) do not differ significantly (sample *A*—left average angle = 63.49° ± 1°, right average angle = 63.88° ± 1°; sample *B*—left average angle = 147.67° ± 1°, right average angle = 147.05° ± 1°), which proves that the conducted measurements were carried out correctly. However, it is worth mentioning the difference in the values obtained for fluids with the addition of silica nanoparticles. In both cases (water or isopropanol), the contact angle is lower than the value for pure liquids by an average of 3°, which indicates a change in the fluid viscosity, and hence lower ductility of the tested fluid. It is worth mentioning that the measurements of the wettability angle were made on carbon tape.

The zeta potential values shown in Table S2 for both water and isopropanol suspensions are about − 60 mV, which indicates high stability and no aggregation in time^23^. This is also confirmed by the lack of major change in aglomerates over a month. It is worth mentioning that the submicron particles suspended in water are smaller than those in isopropanol by about 30–40 nm in terms of mean hydrodynamic diameter and are 185.9 and 212.6 nm, respectively, after a month.

### Proposed multifractal approach

**Figure 1:**
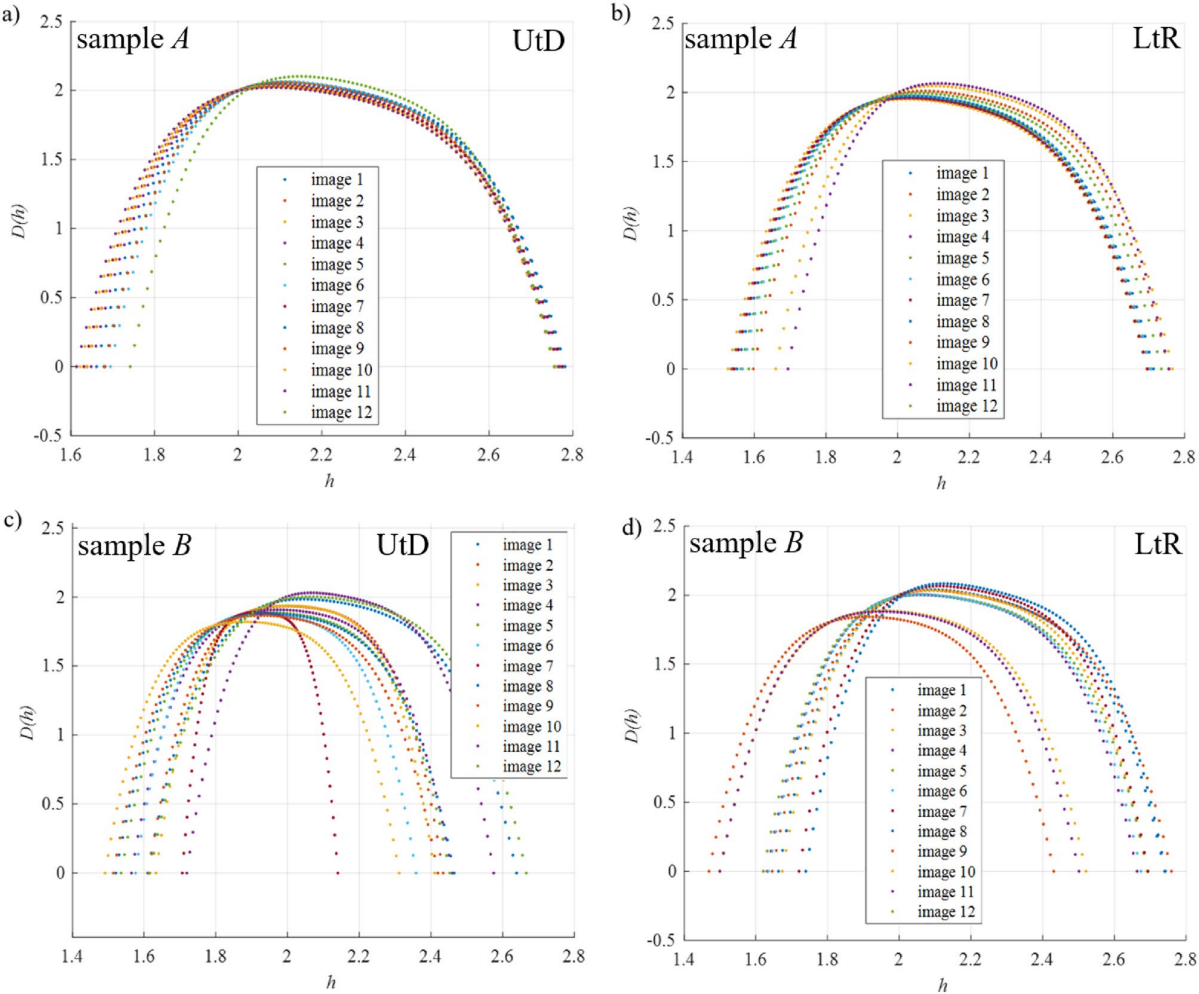
Singularity spectra obtained for sample *A*(SiO_2_ in demineralized water): (**a**) the direction of the UtD (Up to Down) images; (**b**) direction of the LtR (Left to Right) images and sample *B*(SiO_2_ in isopropanol): (**c**) direction of the UtD images; (**d**) direction of the LtR images.

The results of the multifractal analysis folded as consecutive singularity spectra in one plot, for both samples, are presented in Fig. 1. In addition, the values of three characteristic points from the spectra of singularities and the dimension characterizing the width of the multifractal spectrum, which indicates the complexity of the observed process/image have been listed in Table S3.

On Fig. 1 which represents the spectra of singularities for successively taken SEM images for each sample, several characteristic features can be observed. Namely, the multifractal spectra for sample *A* (Fig. 1a,b) are arranged close to each other, even overlapping. Their *h*_*min*_, *h*_*max*_ and *h*_0_ values do not differ significantly from each other in the direction of UtD (Up to Down) and LtR (Left to Right), which suggests correctly distributed nanopowder particles within the liquid throughout the whole sample. In the case of Fig. 1c,d, presenting spectra for sample *B*, significant changes between the shape and position of individual curves can be noticed. This suggests that the fluid was not prepared properly. Thus, there is a significant variation in the size of the observed nanopowder particles and their unregulated distribution on the sample surface.

The numerical values presented in Table S3, allow concluding that: the lower the *h*_*min*_ value, the smaller the nanopowder particles can be observed (better disintegration of the agglomerates during the sonication process), while the higher the *h*_*max*_ value, the more uniform distribution of nanopowder particles in the observed area (lower dispersion of the *h*_*max*_ value also indicates a weaker effect of particle agglomeration). Thus, the value of *h*_*0*_ characterizes the fluid as a whole (as the sum of the particle size and their distribution in the fluid). Therefore, the greater the value in relation to *h*_*min*_ and *h*_*max*_, the better the nanofluid is prepared. Additionally, the width of the multifractal spectrum indicates the complexity of the tested fluid, higher value corresponds to a more uniform nanofluid (smaller nanopowder particles, more evenly distributed on the surface of the sample).

## Discussion

### Standard approach to characterization of nanofluids

**Figure 2:**
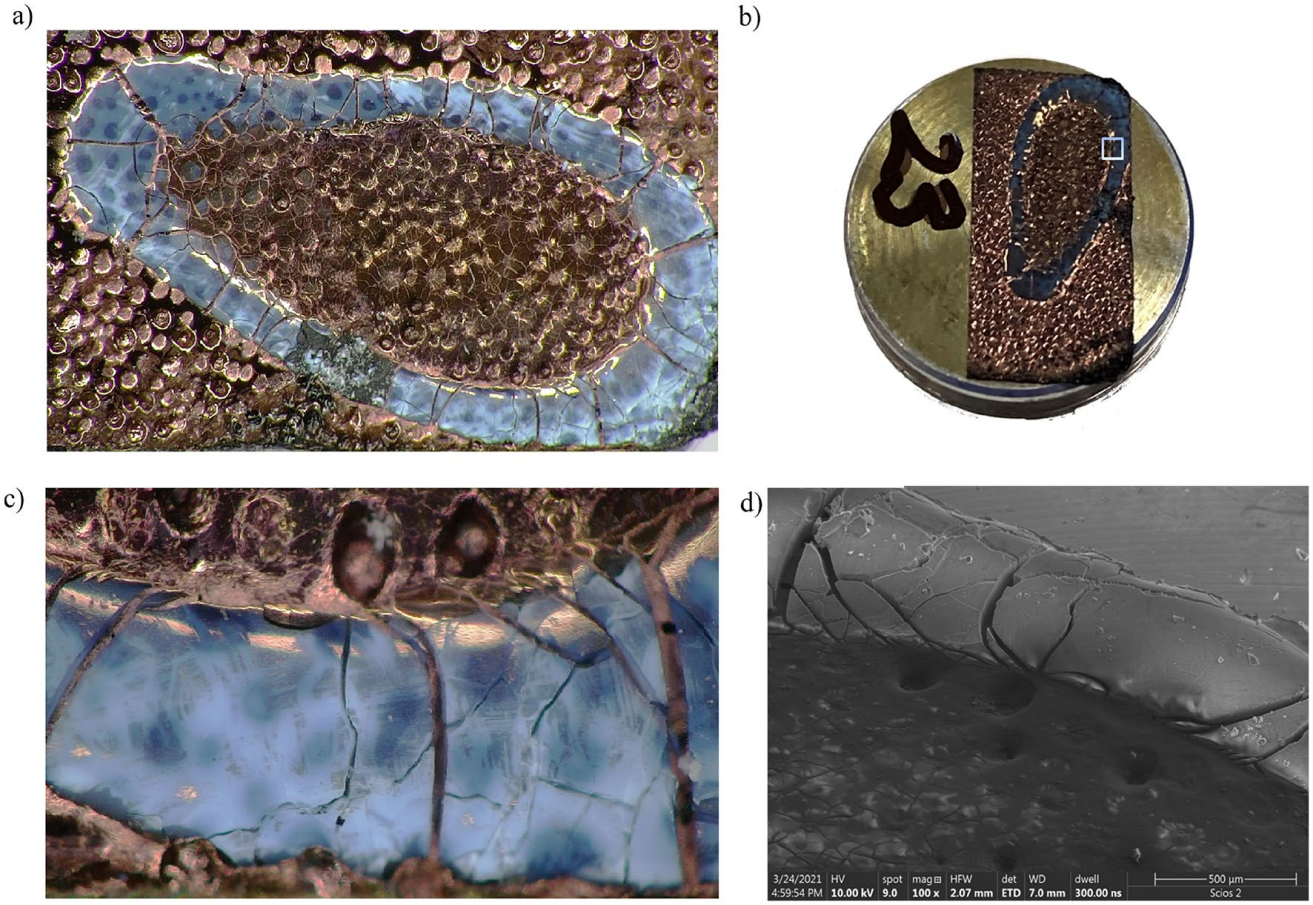
Photos of sample *A*: (**a**) image from optical microscopy; (**b**) macro image of the pin; (**c**) image from optical microscopy of the marked area; (**d**) SEM images of the edge of the sample—powder particles were found both on the edge and inside.

**Figure 3:**
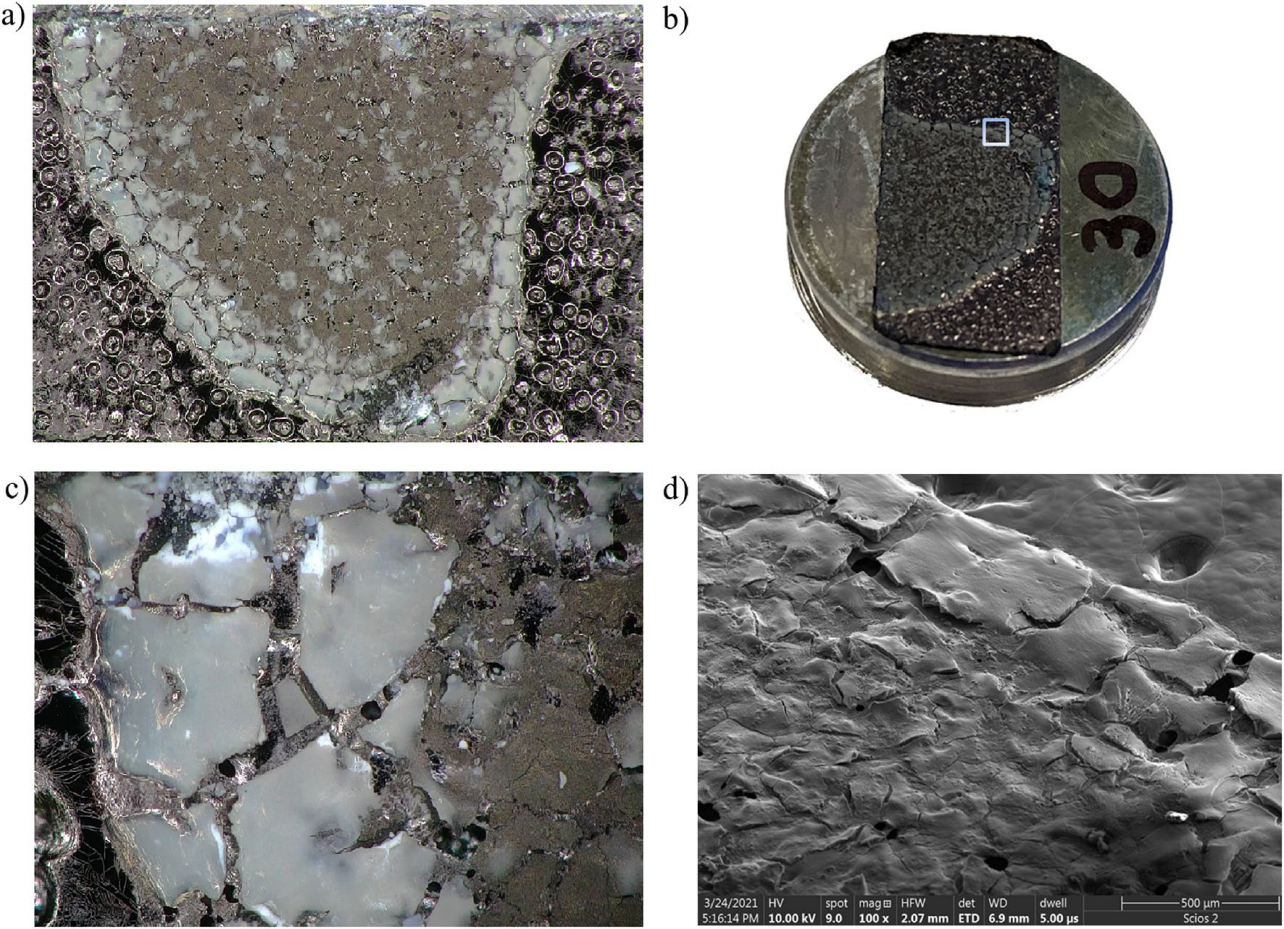
Photos of sample *B*: (**a**) image from optical microscopy; (**b**) macro image of the pin; (**c**) image from optical microscopy of the marked area; (**d**) SEM images of the edge of the sample—powder particles were found both on the edge and inside.

Photograph showing sample *A* (Fig. 2) allow noticing a characteristic ring on the edge of the sample resembling the o-ring like pattern presented in^18^, while sample B (Fig. 3) does not have such a strongly visible edge (it is visible but is irregular and fades away in places), indicating a different character of the evaporation process.

**Figure 4:**
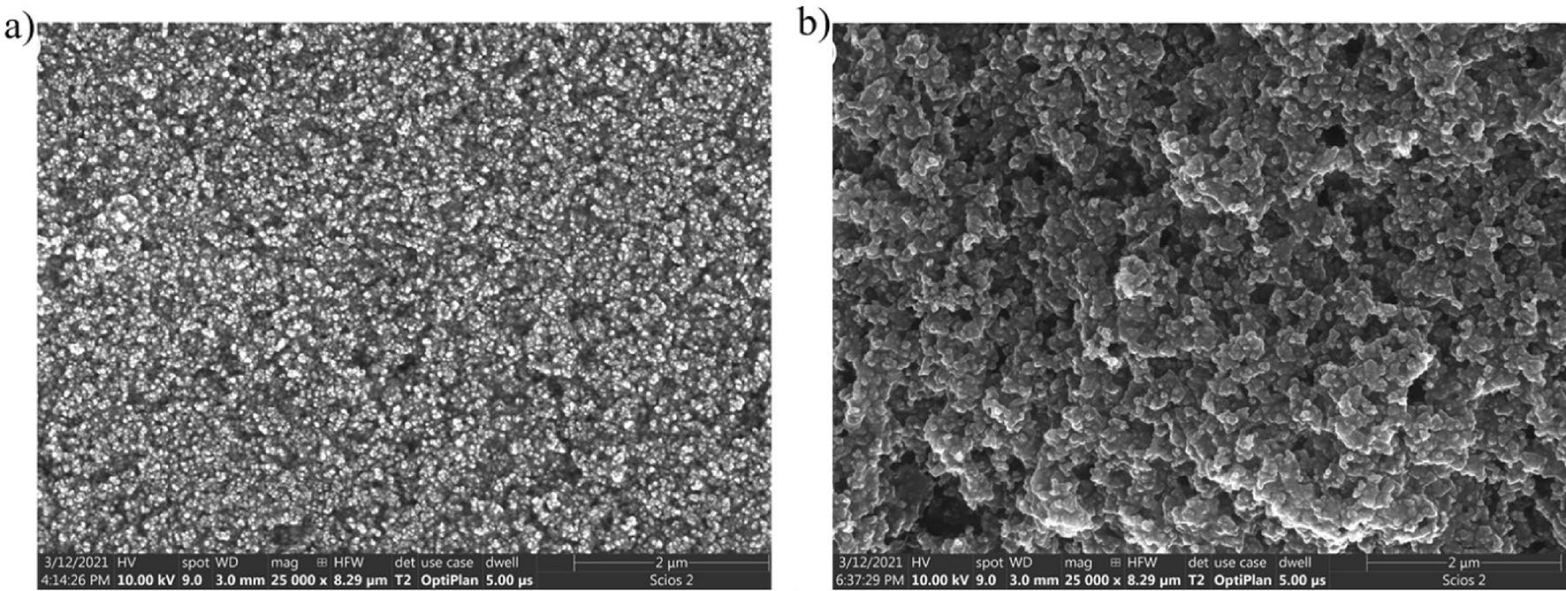
SEM images of (**a**) sample *A*; (**b**) sample *B*.

The SEM imaging for both samples has been done at a magnification of 25,000 x (Scios 2, ThermoFisher, USA). Exemplary images are shown in Fig. 4—additional SEM images Figs. S12, S14, S17 and S18.

Taking a closer look on the SEM images one can notice that sample *A* (Fig. 4a) and *B* (Fig. 4b) show considerable similarities—both of them show a layer of nanoparticles, more uniform in sample A. Higher magnifications show that sample *A* contains smaller differences in the distribution of nanoparticles—assessed based on the differences in height, which in turn reflects into the sharpness of the image—difficulties in uniform focus over the entire area can be noticed. Whatmore sample *A* shows homogeneity in terms of particle size, whereas in sample *B* individual nanoparticles located on larger "flakes" (agglomerates) can be observed. Sample *B* shows voids within layers, which are not observed in sample *A*. Observation of the same samples under higher magnification better reflects the nature of the formation of algomerates in the case of sample *B*. The size of the particles in the TEM images allow us to say that the particles are below 100 nm.

**Figure 5:**
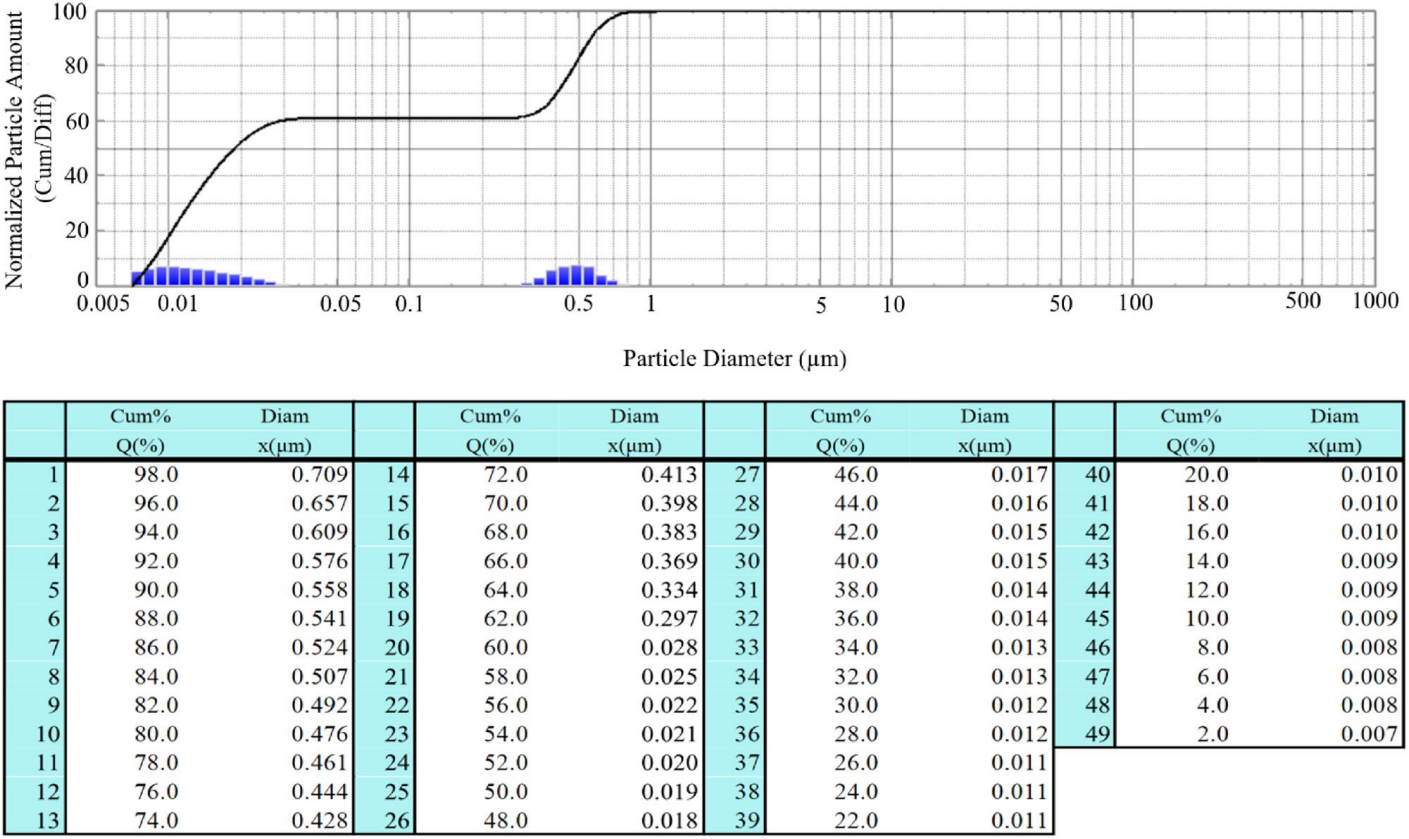
Silica particle size distribution for the sample water + SiO_2_ immediately after the preparation of the liquid.

**Figure 6:**
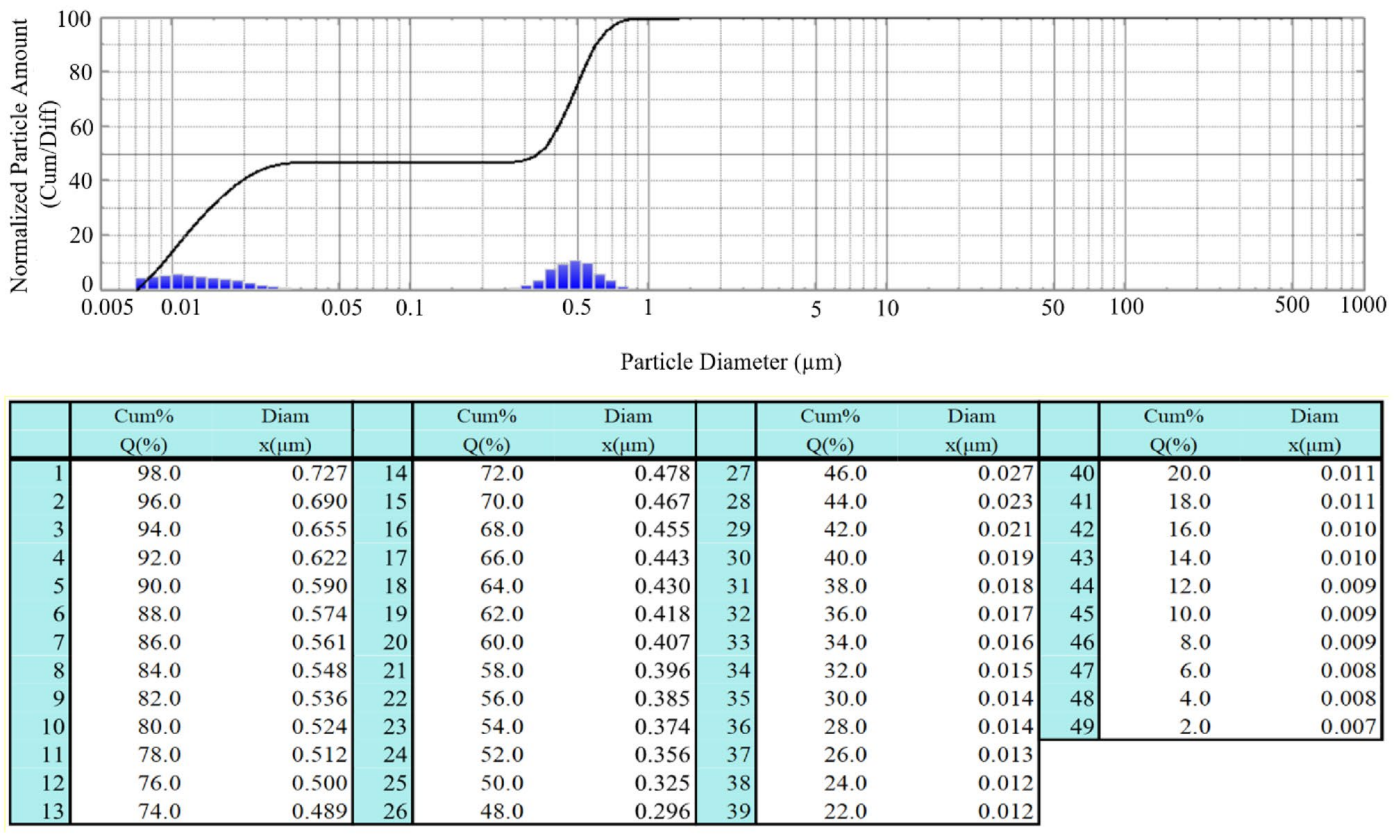
Silica particle size distribution for the sample water + SiO_2_ 24 h after the preparation of the liquid.

Figures 5 and 6 present the results of nanofluid particle size measurements analysis for two cases: (1) concerns liquid immediately after the sonication process in an ultrasonic cleaner, (2) concerns the same liquid that was left for 24 h to determine the stability of the obtained liquid with respect to agglomerates formation. When analyzing the presented data, attention should be paid to two main parameters describing the tests: the measured particle size (diam) and the percentage of particles below this micron size (Q%). In the case of nanofluid that was tested immediately after its preparation, we see that the obtained results indicate silica particles smaller than 0.709 µm—Q% at the level of 98%. The graph also shows numerous particles less than 0.05 µm in size. In the second graph (nanofluid after 24 h), the value of the silica size for Q% at 98% increased to 0.727 µm, which suggests formation of small agglomerates. Other measured powder sizes are similar, which in turn suggests that the obtained fluid is stable. As presented in^24^, a stable nanoliquid is theoretically possible as long as the particles remain small enough (~ 100 nm).

The pH values for both samples were: 5.2 pH for sample *A* and 4.4 pH for sample *B*. According to^13^ the higher the pH value the better the stability of the nanofluid. Whatmore the zeta potential also should be greater for higher pH values.

Zeta potential (ZP) is the potential at the sliding border, i.e., at the point of contact between the solid and the electrolyte solution. The ZP determines the stability and the possibility of aggregation of nanoparticles (NPs), which may be crucial for further applications. The values can be both positive and negative and are expressed in mV. Higher absolute values of the zeta potential are associated with a more stable nanofluid. It is assumed that NPs are stable above + 30 mV or below − 30 mV and aggregation occurs in the range from − 5 to + 5 mV^24^. Table S2 shows the development of the zeta potential of silica suspensions and hydrodynamic diameter of particles over time.

**Figure 7:**
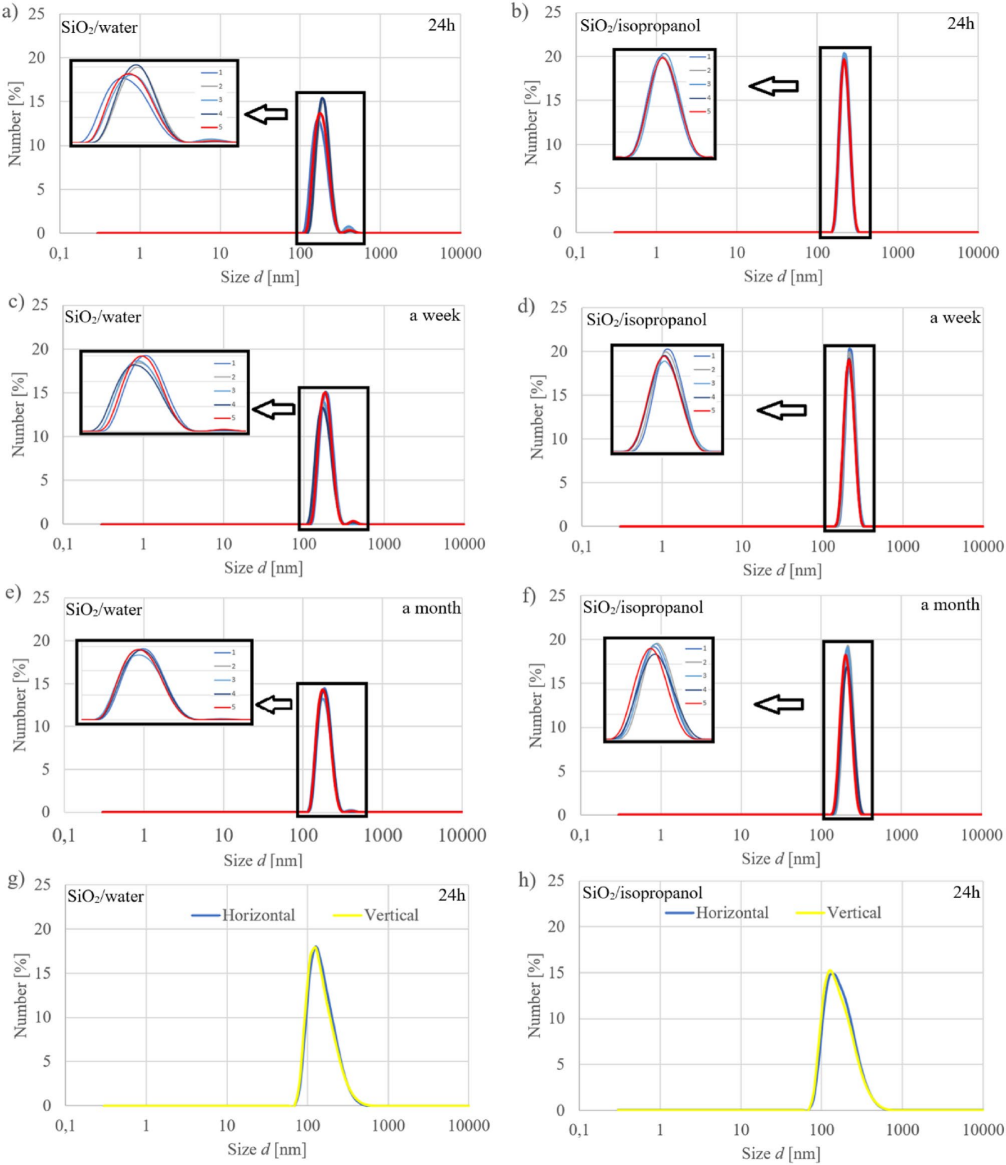
Particle size spectra recorded with MADLS.

The DLS study showed that the second population can be observed in the spectra of silica in water after 24 h, a week, and a month. In isopropanol after 24 h. It accounts for about 1% of the total, and its size is twice the size of the particles. In addition, the strongly negative value of the zeta potential suggests that these are agglomerates resulting from the process of their production, and not the process of preparing suspensions. Additionally, the DLS analyses carried out with the use of horizontal and vertical polarizers made it possible to state that the suspended silica particles are spherical, as evidenced by the overlapping of spectra recorded for both polarizers, which was presented on Fig. 7.

### A new method for the characterization of nanofluids

**Figure 8:**
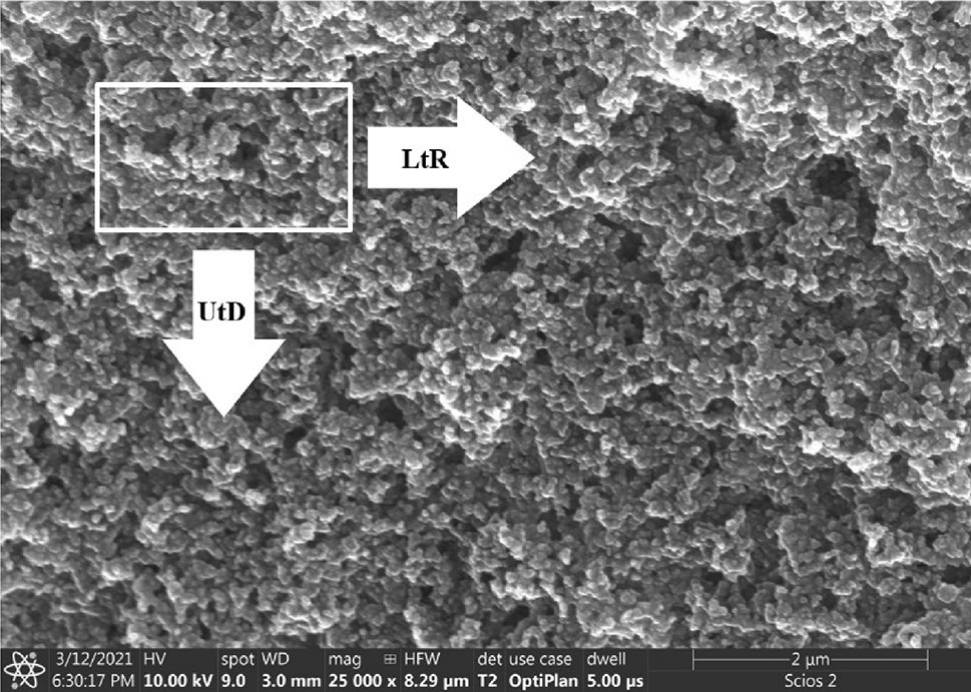
Scheme of the imaging process during SEM observations (sample *B*).

The images analyzed with the nonlinear algorithm were mainly based on moving the seen area (ROI—Region Of Interest) over the entire area of the sample in two schemes: from the left to the right edge of the sample (marked as LtR-left to right) and from top to bottom of the sample (labeled as UtD-up to down)—Fig. 8. Based on obtained images, it was possible to determine the quality of the obtained fluid by analyzing the surface of the sample.

The three characteristic points, in Fig. S11, have been marked with symbols: "cross", "*circle*" and "*square*". The first and third symbols refer to changes taking place in the system, depending on the point of observation, respectively locally or globally in scale. These points were determined using the polynomial approximation method (average value R^2^ = 0.98 for all samples—this proves a very good adjustment of the curve to the results and could be the basis for further analysis). The maximum value in the spectrum, marked with a circle, determines the sum of two other points and identifies the entire process (*h*_0_)^25^. The proposed approach allows to determine singularity spectrum for a single image so the method will be applied to analyze a certain selected area of each sample. It should be noted here that the algorithm of multifractal analysis depends largely on the quality of the analyzed image. Well, in the case of larger magnifications, the image quality deteriorated, areas with voids around which larger agglomerates were formed effectively prevented the full spectrum of singularities from being obtained, thus preventing the complete analysis of the multifractal spectrum (see Figs. S13, S15 and S16—only for those magnifications it was possible to calculate the spectras).

By relying only on single methods (pH value, zeta potential, MADLS ect.) of characterizing nanofluids, we are not able to capture all the elements influencing the quality of the nanofluid (each technique has its own limitations), where using multifractal analysis we analyze a signal that in its structure (history) contains every detail constituting a fundamental particle giving a ready-made product in the form of a nanofluid. To be more precise, in non-linear analysis we do not limit the information that characterizes the tested fluid or the technique that describes it.

## Conclusion

The nanofluids are used in more and more applications, in various areas of life and industrial sectors. Hence, the evaluation of the preparation process and the quality of the fluids obtained is of key importance. The presented results are preliminary and show the possibility of the application of multifractal analysis for evaluation of the quality of prepared nanofluid. Authors strongly believe that proposed approach can be considered as a theoretical and may provide support for/be complementary to the experimental methods.

The samples obtained as a result of evaporation resemble those reported in the literature, while the presented tests require additional trials and analyzes to further describe both the nanofluid preparation process as well as the samples of dried droplets.

The research carried out on the obtained nanofluid shows that the applied approach to the SiO_2_ powder allows to obtain a stable (in terms of the powder particle size at different time intervals from the moment of obtaining the nanofluid—Zeta potential, particle analyzer, MADLS) fluid. Further research will concern other fluid and nanoparticle compositions that allow to obtain different fluid properties (density, viscosity, thermal conductivity, etc.) and the use of multifractal analysis for both photos of traces left by a dried drop of nanofluid and macro/microscopic photos of the prepared nanofluid in a liquid form.

In the multifractal diagram, three characteristic points can be determined, the leftmost one in the case of nanolfluids characterizes the size of the particles suspended in the fluid—the lower the value of *h*_*min*_, the smaller the particles appear. The rightmost point describes the distribution of nanoparticles on the surface. The greater the value of *h*_*max*_, the better the homogenization of the fluid. The maximum value of the multifractal spectrum characterizes the repeatability of the relations found in the entire sample.

The addition of silica powder reduced the wettability of the fluid in both water and isopropanol. The silica increased the fluid density compared to the base fluid, which can be seen from the contact angle values.

## Materials and methods

### Materials

The base material was a commercially available hydrophilic amorphous colloidal silica with a specific surface area (BET) of 200 ± 25 m^2^/g, pH value in 4% dispersion in range 3.7–4.5 and tamped density around 50 g/l. To prepare nanofluids the SiO_2_ nanoparticles (~ 0.01 g) have been suspended in 100 mL of demineralized water (HYDROLAB HLP 5sp, HYDROLAB, Poland) and in 100 ml of 99.5% isopropanol, marked sample *A* and sample *B*, respectively. Sample *A* has been sonicated in common mode, whereas sample *B* in degas mode using Ultrasonic Cleaner (ZX-615FTS, Shanghai ZX Trading Co, LTD, Shanghai) for 60 min according to^26^. Both samples have been sonicated without heating, at 95% power of the device (342 W ultrasonic power).

### Experimental techniques

#### Classic approach

The transmission electron microscopy (TEM) has been used to conduct visualization of silica nanopowder using a scanning TEM (Carl Zeiss Libra 200 MC Cs, Carl Zeiss AG, Oberkochen, Germany), operating at an accelerating voltage of 200 kV (see Fig. S10).

Both nanofluids were analyzed by conventional characterization methods: determination of the contact angle, determination of the zeta potential, pH, and examination with a particle size analyzer. Summing up, this work focuses mainly on two of the five parameters characterizing nanofluids according to^27^: particle and colloidal properties.

The contact angle was measured by a Ossila Contact Angle Goniometer (United Kingdom). Contact angle analyses were performed by the sessile drop technique at room temperature and atmospheric pressure. Ten independent measurements for nanofluids (water and nanosilica, isopropanol and nanosilica) and five independent measurements for pure water and isopropanol were performed for each sample, each with a 15 µL water drop, and the obtained results were averaged to reduce the impact of surface nonuniformity.

The size of silica particles after the sonication process in an ultrasonic cleaner was measured by Nano Particle Size Analyzer SALD-7500nano for the water and silica solution in two stages: (1) immediately after the preparation of the liquid, and (2) 24 h after the preparation of the liquid see Figs. 5 and 6.

The zeta potential and the particle size measurements were conducted by the Multiangle Dynamic Light Scattering (MADLS) and Dynamic Light Scattering (DLS) technique. Zeta potential, MADLS and DLS with horizontal or vertical polarizer were carried out using a Zetasizer Ultra (Malvern Panalytical Ltd., Malvern, UK) equipped with a 10mW helium/neon laser (λ = 633 nm) at 25 °C. The instrument settings were optimized automatically using the ZS XPLORER software (Malvern Panalytical Ltd., Malvern, UK). MADLS measurements were performed after 24 h, a week, and a month after suspension. The particle sizes are expressed as the mean hydrodynamic diameter of 5 measurements.

For the SEM imagining, droplets from Aerosil A200 + water (sample A) and Aerosil A200 + isopropanol (sample B) solution were placed on an aluminum pin disc covered with a double-sided adhesive carbon tape using an automatic pipette Eppendorf Research (volume of the drop: 2 µl). The evaporation process took place at room temperature for 24 h. For imaging at the highest resolution via scanning electron microscopy (FE-SEM) samples were coated with 10 nm of Au using a high-vacuum sputter coater (Compact Coating Unit CCU-010, Safematic, Switzerland). The imaging was carried out with a dual-beam FIB-SEM tool (Scios 2, ThermoFisher, USA) using detectors of secondary electrons: ETD and upper in-lens detector—T2, acceleration voltage of 10 kV for the electrons, 3 mm and 7 mm working distance. Figures 2b and 3b present photos of exemplary SEM pin stubs showing the evaporation effect (which took place without additional heating–free evaporation conditions) on the shape of the dried nanofluid. The magnification of the edges is presented via SEM images in Figs. 2d and 3d, respectively (~ 100×).

#### A new approach with the use of multifractals

The multifractal approach opens possibilities for the visualization of materials as a heterogeneous system with all aspects as subsets of the fundamental elements. The result of this analysis presents a multifractal spectrum that describes the dimension of a fractal subset of function points. To obtain an adaptive form of division both in time and space, the result of which is the spectre of singularities, the Legendre transformation (*τ*(*q*)) is used. This transformation defines the relationship between itself and the global spectrum of singularities *D*(*h*)^28^:

1 $$ D(h) = qh(q) - \tau (q), $$

2 $$ h(q) = \frac{d\tau (q)}{{dq}}, $$

where *h*(*q*) is the Hölder exponent for the moment *q*. Negative values of the moment *q* refer to weak exponents, where positive—analogous to the strong ones. The spectrum itself can be written as:

3 $$ D\left( h \right) = \lim_{l \to 0} \frac{{\sum\nolimits_{i = 1}^{N\left( l \right)} {\mu_{i} \left( {q,l} \right)\ln \left[ {\mu_{i} \left( {q,l} \right)} \right]} }}{\ln l}, $$

where *D*(*h*) is the function of moments *q*, *µ*_*i*_(*q*, *l*) is a normalized measure as *q*^*th*^ moment of mass probability *P*_*i*_(*l*) where to estimate multifractal properties over a small interval of scales a constant range of *l* is taken advantage of^29^:

4 $$ \mu_{i} \left( {q,l} \right) = \frac{{P_{i}^{q} \left( l \right)}}{{\sum\nolimits_{i = 1}^{N\left( l \right)} {P_{i}^{q} \left( l \right)} }}. $$

The explementary result of the multifractal analysis algorithm of SEM images in the form of a singularity spectrum for sample *A* is shown in Fig. S9.

The polynomial approximation method allows to determine three characteristic points on the graph (see Fig. S11).

## Supplementary Information

Supplementary Information.

## Data Availability

The datasets used and/or analysed during the current study available from the corresponding author on reasonable request.
