# Supplementary material for: Characterization of nanofluids using multifractal analysis of a liquid droplet trace
Source: Sci Rep. 2022 Jun 30;12:11111. doi: 10.1038/s41598-022-15402-4 (PMC9247165; doi:10.1038/s41598-022-15402-4)
Supplement: Supplementary file 1 — Supplementary Information. [file 41598_2022_15402_MOESM1_ESM.docx]

**Supplementary Information**

**CHARACTERIZATION OF NANOFLUIDS USING MULTIFRACTAL ANALYSIS OF A LIQUID DROPLET TRACE**

Augustyniak J., Zgłobicka I., Kurzydłowski K., Misiak P., Wilczewska A. Z.,
Jürgen Gluch, Zhongquan Liao, Perkowski D. M.

**Figures**

Fig. S9. Example of multifractal spectrum calculated for a single SEM image (sample A).


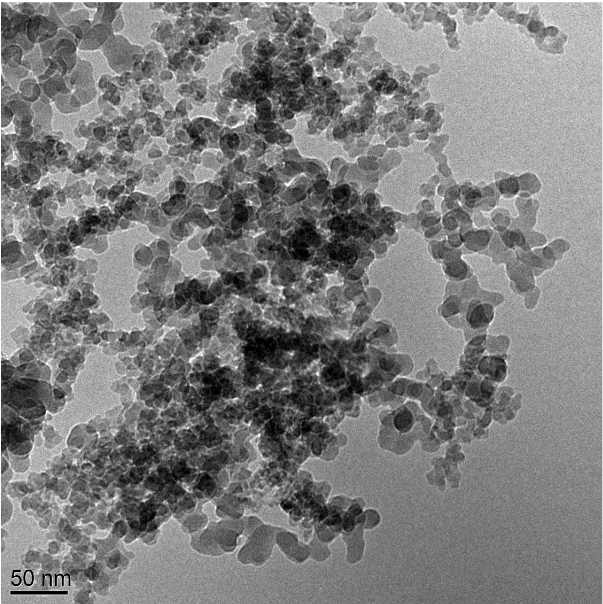

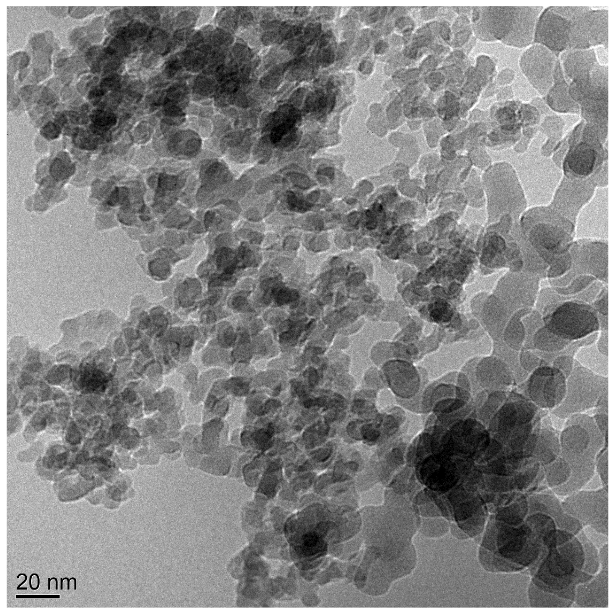


**Fig. S10.** TEM images of raw SiO2 powder

Fig. S11. Multifractal spectrum for a single SEM image with three characteristic points: *h_min_*, *h_max_* and *h_0_* (sample *A*).


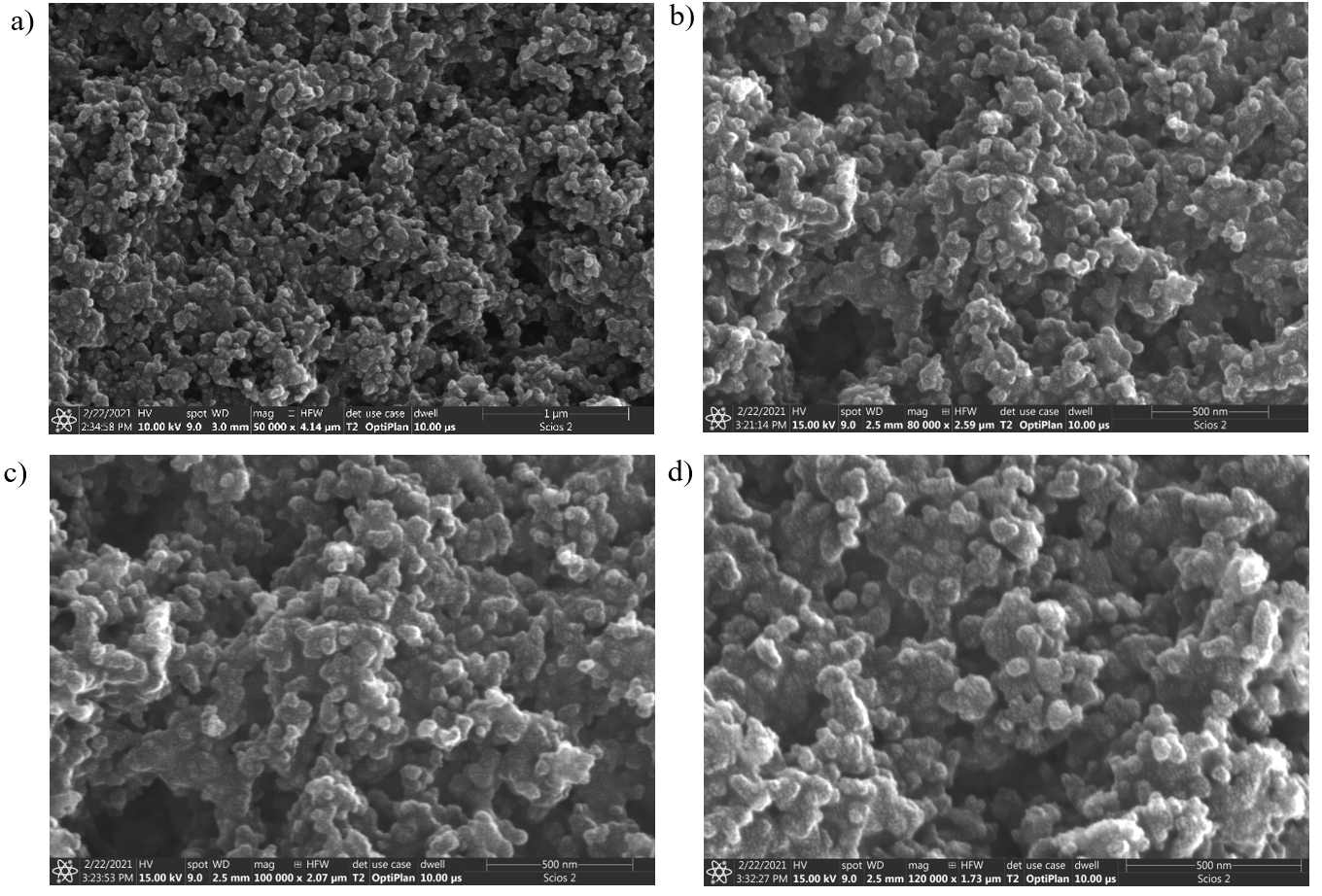


Fig. S12. SEM images of sample *B* for higher magnifications: a) 50000x; b) 80000x; c) 100000x; d) 120000x.

Fig. S13. Multifractal spectrum for a single SEM image (50000x magnification) with three characteristic points: *h_min_*, *h_max_* and *h_0_* (sample *B*).


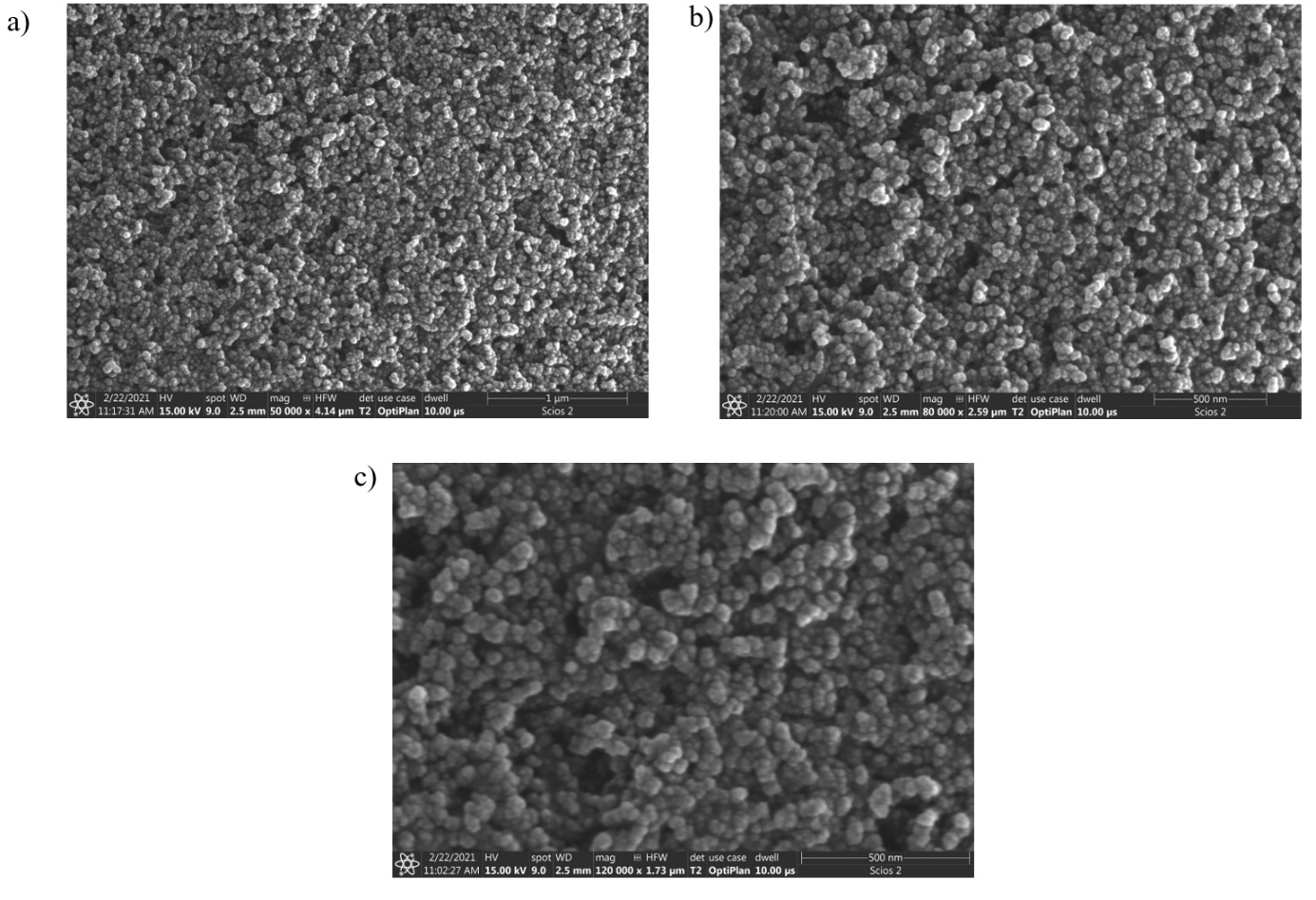


Fig. S14. SEM images of sample *A* for higher magnifications: a) 50000x; b) 80000x; c) 100000x.

Fig. S15. Multifractal spectrum for a single SEM image (50000x magnification) with three characteristic points: *h_min_*, *h_max_* and *h_0_* (sample *A*).

Fig. S16. Multifractal spectrum for a single SEM image (80000x magnification) with three characteristic points: *h_min_*, *h_max_* and *h_0_* (sample *A*).


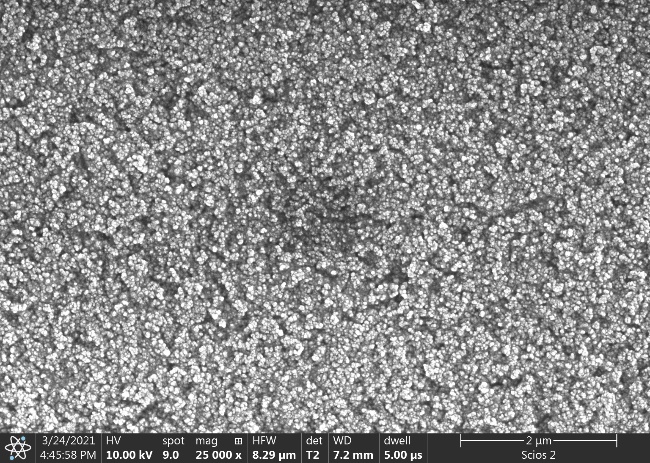

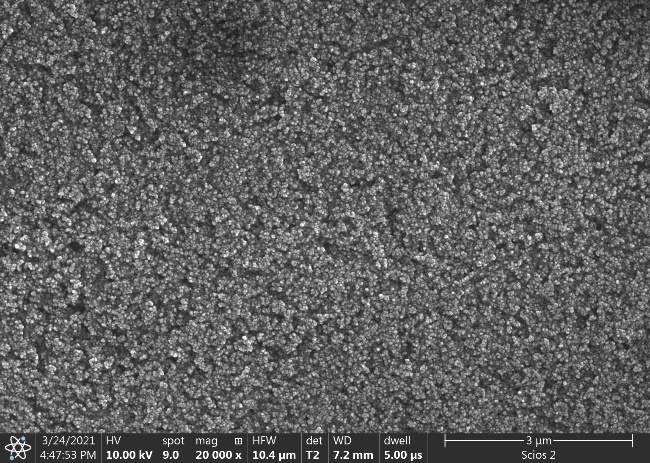


Fig. S17. SEM images of sample A (25000x and 20000x magnification) from different places on the stub.


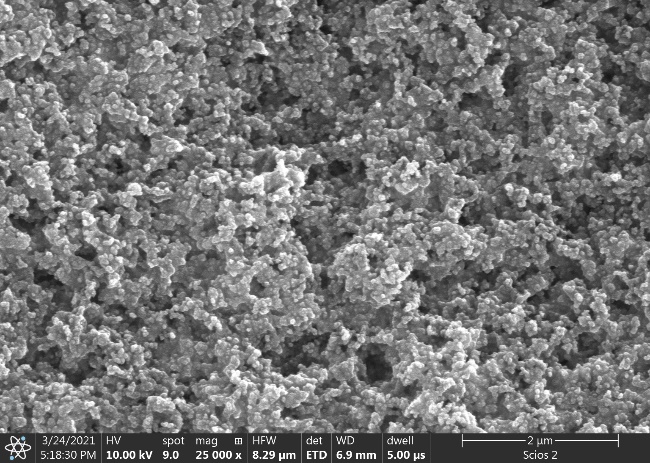


Fig. S18. SEM images of sample B (25000x magnification) from a different place on the stub.

**Tables**

Tab. S1. Compilation of the contact angle for two samples: A and B.

| Angle/specimen | sample *A* | water | sample *B* | isopropanol |
| --- | --- | --- | --- | --- |
| Left average angle | 63.49°±1° | 66.68°±1° | 147.67°±1° | 151.81°±1° |
| Right average angle | 63.88°±1° | 67.02°±1° | 147.05°±1° | 151.15°±1° |

**Tab. S2.** Zeta potential of silica suspensions and hydrodynamic diameter of particles in time.

| medium | ζ potential | Size from MADLS | | |
| --- | --- | --- | --- | --- |
|  | [mV] | [d, nm] | | |
|  |  | after 24 h | after a week | after a month |
| water | -60.89 ± 3.93 | 187.8 ± 7.7 | 186.4 ± 4.9 | 185.9 ± 2.5 |
| isopropanol | -57.29 ± 1.83 | 221.6 ± 2.5 | 219.6 ± 3.6 | 212.6 ± 5.2 |

**Tab. S3.** Summary of the most important values obtained based on multifractal analysis.

| Sample / method | Mean ***h_min_*** value | Mean ***h_max_*** value | Mean ***h_0_*** value | Mean ***h_max_*-*h_min_*** value |
| --- | --- | --- | --- | --- |
| *A* / LtR | 1.575 | 2.719 | 1.986 | 1.143 |
| *B* / LtR | 1.618 | 2.646 | 1.989 | 1.028 |
| *A* / UtD | 1.661 | 2.765 | 2.048 | 1.104 |
| *B* / UtD | 1.591 | 2.444 | 1.917 | 0.852 |
